# Mechanisms of the Ammonium Sulfate Roasting of Spent Lithium‐Ion Batteries

**DOI:** 10.1002/gch2.202200053

**Published:** 2022-11-18

**Authors:** Xin Qu, Yiqi Tang, Mengting Li, DongXu Liu, Shuaibo Gao, Huayi Yin

**Affiliations:** ^1^ School of Resource and Environmental Sciences Wuhan University 299 Bayi Road, Wuchang District Wuhan 430072 P. R. China; ^2^ Key Laboratory for Ecological Metallurgy of Multimetallic Mineral of Ministry of Education School of Metallurgy Northeastern University Shenyang 110819 P. R. China

**Keywords:** ammonium sulfate roasting, mechanisms, recovering, Šatava–Šesták method, spent lithium‐ion batteries

## Abstract

Ammonium sulfate ((NH_4_)_2_SO_4_) assisted roasting has been proven to be an effective way to convert spent lithium‐ion battery cathodes to water‐soluble salts. Herein, thermogravimetric (TG) experiments are performed to analyze the mechanism of the sulfation conversion process. First, the reaction activation energies of the sulfate‐assisted roasting are 88.87 and 95.27 kJ mol^−1^, which are calculated by Kissinger–Akahira–Sunose (KAS) and Flynn–Wall–Ozawa (FWO) methods, respectively. Then, nucleation and growth are determined and verified as the sulfation reaction model by the Šatava–Šesták method. Finally, sub‐reactions of the sulfation process are investigated and reaction controlling mechanisms are determined by the contribution of sub‐reaction. Based on the thermogravimetric analysis, the phase boundary reaction is found to dominate in the initial step of the roasting process (α < 0.6) while the nucleation reaction controlls the following step (α > 0.6), agreeing well with changing trend of activation energy. Overall, thermogravimetric analysis is a general way to study the mechanism of the various roasting processes.

## Graphical abstract

Thermogravimetric experiments are performed to analyze the mechanism of the sulfation conversion process. First, the reaction activation energies of sulfate‐assisted roasting are calculated using two different models. Then, nucleation and growth are determined and verified as the sulfation reaction model by the Šatava‐Šesták method. Finally, sub‐reactions of the sulfation process are investigated and reaction controlling mechanisms are determined.

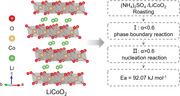

## 1 Introduction

Since the industrial revolution, rapid economic growth has been inextricably linked with the massive consumption of energy. However, the current global energy problem has already become the main and urgent obstacle to promoting economic and sustainable development.^[^ ^1^, ^2^ ^]^ For example, lithium‐ion batteries (LIBs) were largely produced and used in two major areas: consumer electronics and electric vehicles (EV) due to their excellent properties including low self‐discharge rate,^[^ ^3^, ^4^, ^5^ ^]^ wide operating temperature range,^[^ ^6^, ^7^ ^]^ etc. At the same time, the quantity of spent LIBs has exploded due to their short lifespan and fast replacement.^[^ ^8^, ^9^ ^]^ According to Gavin et al.,^[^ ^10^ ^]^ about 25 thousand tones and half a million cubic meters of unprocessed pack spent LIBs will be retired from EVs at 2025. Besides, the heavy metal elements in the spent LIBs, such as Ni, Co, and Mn, will have a bad influence on the environment and human‐health.^[^ ^11^, ^12^ ^]^ On the other hand, the contents of metal elements in the spent LIBs are much higher than their contents in the crust. Consequently, the treatment and metal recovery from the spent LIBs has become imperative and significant from the standpoint of both resource preservation and environmental protection.^[^ ^13^ ^]^

At present, great efforts have been made to recycle spent LIBs. There are three typical routes including pyrometallurgy, hydrometallurgy, and biometallurgy.^[^ ^14^, ^15^, ^16^ ^]^ The hydrometallurgical methods always maintain high efficiency and low energy consumption.^[^ ^17^, ^18^ ^]^ But there is more work to simplify the recovering process, reduce the use of acid‐base, and decrease the production of secondary pollution. For traditional pyrometallurgical methods, simple processes and operations are the reasons why they can be industrialized widely.^[^ ^19^, ^20^, ^21^ ^]^ In contrast, biometallurgical methods still cannot attract more attention due to low recovery rates and strict environment of bacteria.^[^ ^22^, ^23^ ^]^ However, low efficiency and high energy consumption can hinder the development of pyrometallurgy method. According to early Xu's study,^[^ ^24^ ^]^ only 80% of lithium elements can be recycled by a carbothermic pyrolysis approach in 973 K. Hence, more attention has been paid to a hybrid of pyro‐hydro methods owing to their high efficiency, low energy consumption, and less secondary pollution.

Recently, salt‐assisted roasting routes have attracted more and more eyes to recycling cathode materials from spent LIBs instead of mineral extraction. The reason is that the crystal structures of lithium transition metal oxide can be broken down by ion‐exchange reactions at a lower temperature (below 1073 K).^[^ ^25^, ^26^, ^27^ ^]^ Fan et al.^[^ ^17^ ^]^ and Qu et al.^[^ ^20^ ^]^ reported an environmental‐friendly NH_4_Cl‐assisted roasting process to convert LiCoO_2_(LCO) to water‐soluble chloride salt at 623 K. In our previous work, (NH_4_)_2_SO_4_ was chosen as a reducing agent to convert Li, Co, Ni, and Mn in cathode materials into the corresponding sulfate to recover key metal elements. Recovery rates of metal elements could reach over 98% between 623 and 673 K for 2 h, and the least mass ratio of (NH_4_)_2_SO_4_/(cathode materials) was 3.5:1.^[^ ^7^, ^28^ ^]^ Moreover, the whole recycling process did not introduce other metal element impurities which need to be separated by the following steps. In a word, salt‐assisted roasting has been regarded as a promising method to replace traditional pyrometallurgical methods. However, there are still more efforts that need to be devoted to realizing the practical and industrialized application of recycling spent LIBs. Note that, the focus of most research so far has been on process parameters rather than mechanisms. Understanding the roasting mechanism in depth is important to the industrialized application. Besides, kinetic study on salt‐assisted roasting has a great significance for increasing the reaction rates, controlling reaction processes, and improving the recovery efficiency.^[^ ^29^ ^]^ Therefore, the investigation of mechanisms is becoming more necessary and worthwhile for the further development of salt‐assisted roasting technology for spent LIBs.

In this paper, kinetics and mechanism of (NH_4_)_2_SO_4_‐assisted roasting in the air at the heating rate of 5, 10, 15, and 20 K min^−1^ were investigated by thermogravimetry (TG) analysis and iso‐conversational methods. Three popular model‐free methods: Friedman, Kissinger–Akahira–Sunose (KAS), Flynn–Wall–Ozawa (FWO) methods, and a model‐fitting method (CR method) were employed to conduct the kinetic analysis. Finally, the accuracy and applicability of the values of the calculated kinetic parameters were evaluated. Based on the obtained values of kinetic parameters, the thermodynamic parameters were calculated and analyzed to make a better understanding of sulfation roasting process. Detailed data processing and analyzing method are shown in Figures S1–S2 and Equations S1–S7, Supporting Information.

## 2 Results and Discussions

### 2.1 Sulfation Reaction Determination

**Figure 1:**
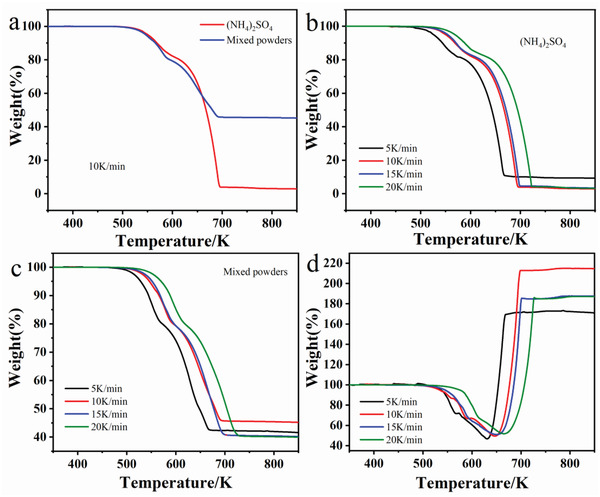
TGA curves of a) mixed powders with a heating rate of 10 K min^−1^ and (NH_4_)_2_SO_4_, b) pure (NH_4_)_2_SO_4_, c) mixed powders, and d) LCO sulfation with different heating rates (atmosphere: Air, (NH_4_)_2_SO_4_/LCO = 4:1 wt/wt).

TG test was arranged to determine sulfation reaction. The results are shown in **Figure** **1**. As shown in Figure 1a, both curves of powders showed substantial weight loss. (NH_4_)_2_SO_4_ decomposed completely at approximately 695 K. In contrast, mixed powders maintained 46% of the initial mass due to the decomposition of (NH_4_)_2_SO_4_ and sulfation conversion of LCO. To investigate the mechanism of sulfation conversion of LCO, different heating rates including 5, 10, 15, and 20 K min^−1^ were selected for thermogravimetric analysis (TGA) experiments of pure (NH_4_)_2_SO_4_ and mixed powders as shown in Figure 1b,c. Obviously, only a weight loss process was observed at a small temperature interval. In other words, both the decomposition of (NH_4_)_2_SO_4_ and sulfation conversion were a rapid process. Therefore, data processing was conducted to define the real weight change curves of LCO. The detailed processing and analyzing processes are shown in Supporting Information. As shown in Figure 1d, there was only a peak including a weight loss and increasing process. Ultimately the sulfation process of LCO was a weight‐increasing process. However, it was an obvious weight‐loss process when the sulfation reaction is shown in Equation (1). Therefore, the gas‐solid reaction is one of the main reactions of the sulfation process. According to literature,^[^ ^30^ ^]^ the decomposition products of (NH_4_)_2_SO_4_ were NH_3_(g), N_2_(g), SO_2_(g), and H_2_O(g) as Equation (2). Herein, the sulfation reaction of the weight‐increasing process should be shown in Equation (3), and the theoretical increment was 224.49%. Besides, the results also indicated that the whole sulfation reaction can be divided into two parts: decomposition of (NH_4_)_2_SO_4_ and sulfation conversion of LCO. This was in line with the results of our previous work.^[^ ^28^ ^]^ It should be noted that double salt ((NH_4_)_2_Co(SO_4_)_2_) decomposed entirely as the temperature rose.

(1) $$\begin{array}{c} \begin{array}{l} {\text{LiCoO}}_{2}+3/2{\left({\text{NH}}_{4}\right)}_{2}{\text{SO}}_{4}\to {\text{CoSO}}_{4}+1/2{\text{Li}}_{2}{\text{SO}}_{4}\\ \;\;\;+1/6N_{2}\left(g\right)+8/3{\text{NH}}_{3}\left(g\right)+2H_{2}O\left(g\right) \end{array} \end{array}$$

(2) $$\begin{array}{c} {\left({\text{NH}}_{4}\right)}_{2}{\text{SO}}_{4}\to 4/3{\text{NH}}_{3}\left(g\right)+1/3N_{2}\left(g\right)+{\text{SO}}_{2}\left(g\right)+2H_{2}O\left(g\right) \end{array}$$

(3) $$\begin{array}{c} \begin{array}{l} {\text{LiCoO}}_{2}+1/3N_{2}\left(g\right)+3/2{\text{SO}}_{2}\left(g\right)+H_{2}O\left(g\right)\\ \;\;\;\to 1/2{\text{Li}}_{2}{\text{SO}}_{4}+{\text{CoSO}}_{4}+2/3{\text{NH}}_{3}\left(g\right) \end{array} \end{array}$$

### 2.2 Kinetic Analysis by the Model‐Free Method

**Figure 2:**
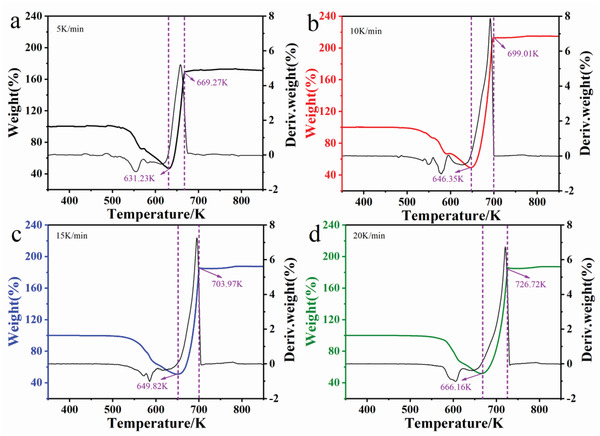
Calculated actual TGA results of reaction substrate under the heating rate of a) 5, b) 10, c) 15, and d) 20 K min^−1^ (atmosphere: Air, (NH_4_)_2_SO_4_/LCO = 4:1 wt/wt).

Referred to the results of Figure 1d, detailed data processing was performed to determine the temperature intervals of LCO sulfation at different heating rates as shown in **Figure** **2**. With the increase in heating rate, the temperature interval of LCO sulfation gradually became larger. And the temperature at which the sulfation conversion started gradually increased. The detailed temperature intervals of LCO sulfation under different heating rates are shown in **Table** **1**, where *T*_1_ and *T*_2_ denote TG curve inflection point. Obviously, the temperature intervals of LCO sulfation became higher and bigger with the heating rate increases, which was called the temperature hysteresis phenomenon. A higher heating rate related to uneven temperature distribution and poor thermal transmission tended to a low reaction efficiency.^[^ ^31^, ^32^ ^]^

**Table 1:** The detailed temperature intervals of LCO sulfation under different heating rates

*i*	*β_i_* [K min^−1^]	*T*_1_[K]	*T*_2_ [K]	Δ*T* [K]
1	5	631.23	669.27	38.04
2	10	646.35	699.01	52.66
3	15	649.82	703.97	54.15
4	20	666.16	726.72	60.56

**Figure 3:**
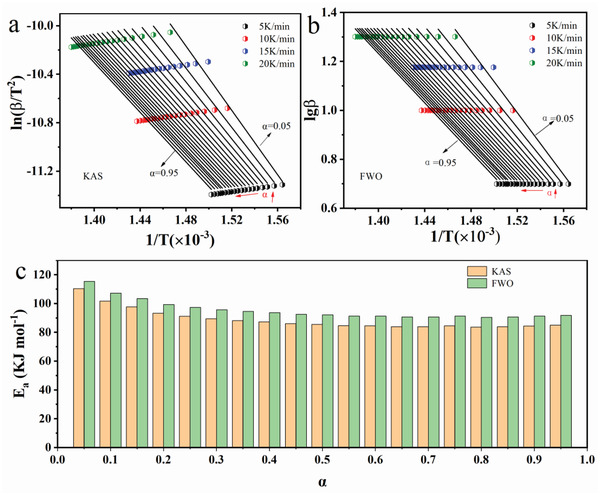
Linear fitting results of a) lg(β/T^2^) versus 1/T and b) lgβ versus 1/T by KAS and FWO methods under different heating rates and reaction fraction, c) calculated activation energies by methods of KAS and FWO (atmosphere: Air, (NH_4_)_2_SO_4_/LCO = 4:1 wt/wt).

**Figure** **3**a,b shows the KAS plots (ln(β/T^2^) against 1/T) and FWO plots (Inβ against 1/T). Based on the results of Table 1, different reaction fractions could be defined by Equation (8). At the given reaction fraction, the average value of activation energy E can be calculated by the slope of KAS and FWO plots. As Figure 3a,b shows, a good and similar fitting occurred between ln(β/T^2^) and 1/T, lnβ, and 1/T. The values of correlation coefficient R^2^ of fitting results at all α were close to 1. In other words, the obtained slope results were reliable. As shown in Figure 3c, the value of E calculated by KAS and FWO methods were almost identical with little difference at the same α. In addition, the value of E decreased at first, then a platform was observed with the increase of reaction fraction. The average values of E in the entire sulfation process were 88.87 (KAS) and 95.27 kJ mol^−1^ (FWO), respectively. The average values of E for KAS and FWO methods was 92.07 kJ mol^−1^ which will be employed as the criteria to determine the dominating reaction model for characterizing the sulfation process by the Šatava–Šesták method.

### 2.3 Kinetic Analysis by Model‐Fitting Method

In the present study, the most suitable reaction model in charge of the sulfation process needs to meet two demands: the *E*_a_ calculated by the Šatava–Šesták method should be close to that obtained by the model‐free methods (92.07 kJ mol^−1^); the value of R2 should be close to 1. Therefore, the activation energy and R2 were obtained by calculating the slope of Šatava–Šesták plot of lg *g*(α) versus 1/T, which was determined by 13 types of common reaction mechanism functions of solid phase, consisting of chemical reaction, diffusion, interface reaction, and nucleation and growth, as listed in Table S1, Supporting Information.

As shown in Table S2, Supporting Information, *E*_a_ was calculated by the Šatava–Šesták method. The values of *E*_a_ were compared with the *E* obtained by the model‐free methods. In those symbols, F1, A2, A3, and A4 were selected because the values of R^2^ were closest to 1. Besides, Equation (4) should be satisfied for the suitable reaction mechanism function. Therefore, *g*(α) = [−ln(1−α)]^1/4^ (A4) may be chosen as the most suitable reaction mechanism function to characterize the sulfation process for the following study, which corresponds to the mechanism of assumed random nucleation and its subsequent growth. The value of R^2^ was 0.9895, and 100.12 kJ mol^−1^ was the closest calculated energy average *E*_a_ value by the Šatava–Šesták method to that by KAS and FWO methods (92.07 kJ mol^−1^). Note that random nucleation meant that a new substance was randomly formed at reactive sites in the reactants lattice. And the growth rate of nucleation may be expressed via the increased formed nuclei radius.^[^ ^33^ ^]^ Avrami–Erofeev model, which combines acceleratory and deceleration rate constants, is generally utilized to express the random nucleation and subsequent growth mechanism.^[^ ^34^ ^]^ In a word, f(α) was the most suitable reaction model in the sulfation process. When α, E, and f(α) were fixed, ln(dα/d*t*), *E*/R*T*, and ln *f*(α) could be calculated, and thus lnA could be deduced by Equation (11). And the value of ln A was 15.59. The detailed reaction model was presented as Equation (5).

(4) $$\begin{array}{c} \left|\frac{E_{0}-E_{a}}{E_{0}}\right|\leq 0.1 \end{array}$$

(5) $$\begin{array}{c} \frac{d\alpha }{dt}=4\exp\left(15.59\right)\exp\left(-\frac{92070}{8.314T}\right)\left(1-\alpha \right){\left[-\ln\left(1-\alpha \right)\right]}^{3/4} \end{array}$$

**Figure 4:**
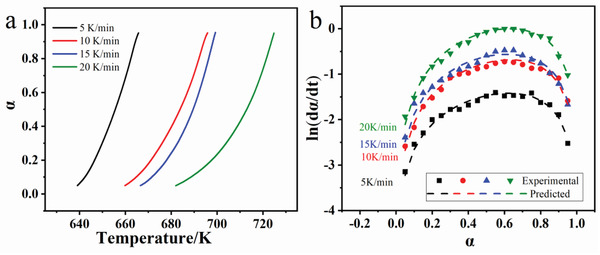
Diagram of a) reaction fraction versus temperature under different heating rates and b) model fitting results of experimental and predicted data.

Then, a necessary model verification was needed to prove the reasonable and availability of the reaction model of the sulfation process. **Figure** **4**a presents the relationship between reaction fraction (α) and temperature based on experimental results. To achieve the same reaction fraction, a higher temperature was required under a higher heating rate. In other words, a high heating rate corresponded to higher energy consumption when other conditions remained the same. Moreover, the predicted ln(dα/d*t*) was calculated by the given α and T by Equation (5) (Figure 4b). And the results showed a good correlation with the method predicted. Therefore, the reaction model as Equation (5) was reasonable and available and was chosen for the following mechanism analysis.

### 2.4 Reaction Controlling Mechanism Analysis

**Figure 5:**
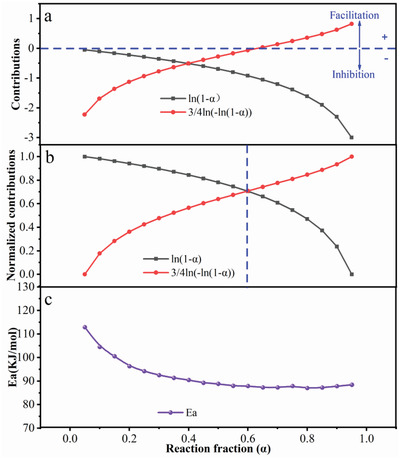
a) Contributions and b) normalized contribution of two sub‐reaction mechanisms to the whole sulfation reaction under different reaction fractions; c) the calculated activation energy under different reaction fractions.

Based on the above discussion, nucleation and growth were the sulfation reaction model which also could be presented as Equation (6). Herein, the item of ln(1−α) and 3/4ln[−ln(1−α)] represented the phase boundary reaction mechanism and nucleation reaction mechanism, respectively.^[^ ^35^ ^]^ Hence, the contributions of the above‐mentioned reaction mechanism were further calculated to analyze the controlling mechanism in every stage of the sulfation process. And results are shown in **Figure** **5**a. The positive value of contributions indicated that the sub‐reaction could facilitate the sulfation reaction. In contrast, the negative value meant that the sulfation reaction was suppressed. However, a simple computation (addition or subtraction) of the contribution values could not directly determine the overall effect of different sub‐reactions. Therefore, determining the key sub‐reaction in the sulfation process at the given condition was a solution. For that, the normalization of the contributions was necessary for distinguishing the key sub‐reaction at the corresponding stage, and the results were presented in Figure 5b.

(6) $$\begin{array}{c} \ln\frac{d\alpha }{dt}+\frac{92070}{8.314T}=\ln\left(1-\alpha \right)+3/4\ln\left[-\ln\left(1-\alpha \right)\right]+15.59+\ln4 \end{array}$$

First, the normalized contributions of two sub‐reactions (Figure 5b) were used to determine the key sub‐reactions at a different reaction fraction. Then, the trends of key sub‐reaction (Figure 5a) could determine the effects of two sub‐reactions for the whole sulfation reaction, which could help figure out the reaction controlling mechanism at specified steps. Based on this, the reaction controlling mechanism of the whole sulfation converting process could be divided into two stages:1) phase boundary reaction controlling step (0.05 < α < 0.6) and 2) nucleation reaction controlling step (0.6 < α < 0.95). In stage 1, the phase boundary reaction mechanism played a leading role and its facilitation weakened with the new crystal nucleation, which led to the decrease of activation energy in Figure 5c. Besides, the phase interface between LCO and gas which was generated by the decomposition of (NH_4_)_2_SO_4_ played a significant part in the sulfation process, causing that phase boundary reaction to occupy control position. In stage 2, the growth of new crystal nucleation gradually dominated as the reaction progresses. At the same time, the phase boundary reaction was still promoting the overall sulfation process, and the activation energy remained basically unchanged under the combined effects.

### 2.5 Theoretical Background and Sulfation Roasting Transformation Analysis

#### 2.5.1 Kinetic Theory

TG provides an ideal environment for the degradation of the small solid sample in which the atmosphere and heating rates can be well controlled.^[^ ^36^, ^37^ ^]^ The solid reaction rate during the decomposition can be written as Equation (7):

(7) $$\begin{array}{c} \frac{d\alpha }{dt}=k\left(T\right)f\left(\alpha \right) \end{array}$$

Where α denotes conversion rate according to TGA results; *t* is time, f(α) represents the different reaction model (or mechanism function), *g*(α) is the integral function and k(T) denotes a constant with temperature T, α, *g*(α) and k(T) can be defined as follows:

(8) $$\begin{array}{c} \alpha =\frac{m_{0}-m_{t}}{m_{0}-m_{\infty }} \end{array}$$

(9) $$\begin{array}{c} k\left(T\right)=Aexp\left(\frac{-E_{a}}{RT}\right) \end{array}$$

(10) $$\begin{array}{c} g\left(\alpha \right)=\int_{0}^{\alpha }\frac{d\alpha }{f\left(\alpha \right)} \end{array}$$

There are three types of m (*m*_o_, *m*_t_, *m*_∞_) standing for the sample mass at the initial stage, *t* min, and the end of Part II reaction, respectively. A is the pre‐exponential factor, *E*_a_ represents activation energy, and R means the universal gas constant (8.314 J mol^−1^ K^−1^). Considering the linear relationship between temperature and heating rate (β), and β = d*T*/d*t*, so the detailed kinetic analysis can be substituted as

(11) $$\begin{array}{c} \frac{d\alpha }{dt}=\beta \frac{d\alpha }{dT}=Af\left(\alpha \right)\exp\left(\frac{-E_{a}}{RT}\right) \end{array}$$

In general, the kinetic process is often a multi‐step and complex process instead of a single process.^[^ ^38^ ^]^ And the reaction activation energy is changing during the reaction progress.^[^ ^39^ ^]^ In this study, two popular model‐free methods: Kissinger–Akahira–Sunose (KAS), Flynn–Wall–Ozawa (FWO), and one well‐known model‐fitting method (Šatava–Šesták method), were adopted for the kinetic calculation.

#### 2.5.2 KAS Method

Based on the approximation of p(x) (x = *E*/R*T*) as the first term by Murray and White,^[^ ^40^ ^]^ KAS method (Equation (12)) was developed by Kissinger, Akahira, and Sunose.^[^ ^41^, ^42^ ^]^ After plotting lnβ against 1/T at an equivalent conversion rate, the activation energy *E*_a_ can be obtained by the slope. Besides, pre‐exponential factor A can be calculated if *g*(α) is already known.

(12) $$\begin{array}{c} \ln\left(\ln\frac{\beta }{T^{2}}\right)=\ln\left(\frac{AE_{a}}{Rg\left(\alpha \right)}\right)-\frac{E_{a}}{RT} \end{array}$$

#### 2.5.3 FWO Method

FWO method is an integration method with an equal conversion rate, which is based upon Doyle's approximation of p(x) (x = *E*/R*T*).^[^ ^43^ ^]^ Flynn, Wall, and Ozawa expressed the FWO method in Equation (12).^[^ ^44^, ^45^, ^46^ ^]^ As shown in Equation (13), the activation energy *E*_a_ can be calculated by plotting lgβ against 1/T at any conversion rate α in different heating rates.

(13) $$\begin{array}{c} \mathrm{lg}\beta =\mathrm{lg}\left(\frac{AE_{a}}{Rg\left(\alpha \right)}\right)-2.315-0.4567\frac{E_{a}}{RT} \end{array}$$

#### 2.5.4 Šatava–Šesták Method

Both KSA and FWO methods are model‐free methods. The values of activation energy can be calculated without a known reaction model in advance based upon conversion rate in different heating rates. Therefore, the Šatava–Šesták method^[^ ^47^ ^]^ (Equation (14)) was chosen to calculate the values of kinetic parameters after assuming the reaction model. Besides, lots of common reaction models were chosen for the fitting to gain a reaction model of ammonium sulfate roasting of spent LIBs.

(14) $$\begin{array}{c} \mathrm{lg}g\left(\alpha \right)=\mathrm{lg}(\frac{AE_{a}}{R\beta })-2.315-0.4567\frac{E_{a}}{RT} \end{array}$$

## 3 Conclusions

An in‐depth mechanism of the (NH_4_)_2_SO_4_‐assisted roasting of lithium cobalt oxide (LCO) was studied by thermogravimetric analysis in terms of roasting model and activation energy. The apparent activation energy (*E*_a_ = 92.07 kJ mol^−1^) and pre‐exponential factor (A = exp(15.59)) were obtained by the two model‐free methods such as KAS and FWO. In addition, the mechanism function (f(α) = 4(1−α)[ −ln(1−α)]^3/4^) was determined by the Šatava–Šesták method. Based on the nucleation and growth analysis, the roasting involved two stages: the phase boundary reaction took control in the first stage (α < 0.6), and then the nucleation reaction controlled the following step (α > 0.6). The mechanistic understanding of the sulfation roasting is helpful to design efficient ways to recycle valuable elements from spent LIBs as well as other types of electronic wastes.

## 4 Experimental Section

Spent LCO cathode powders was obtained from a local battery recycling company in Shenyang, Liaoning Province, China. (NH_4_)_2_SO_4_ powders (A.R, 99.5%) were purchased from China National Pharmaceutical Group Co., Ltd. (Sinopharm). The mixed materials were prepared in the mass ratio of (NH_4_)_2_SO_4_/LCO of 4:1. The mixed powder sample was dried in a drying oven at 323 K for 24 h before the TG tests. A TG analyzer (SDTA851E) was utilized to investigate the reaction models and reaction‐controlling mechanisms of mixed powders with a mass of about 10 mg at the heating rate of 5, 10, 15, and 20 K min^−1^ in the air, and the gas flow rate was 150 mL min^−1^. The selected temperature ranged from 373 to 873 K.

## Conflict of Interest

The authors declare no conflict of interest.

## Supporting information

Supporting Information

## Data Availability

The data that support the findings of this study are available in the Supporting Information material of this article.
