# Supplementary material for: Mechanisms of the Ammonium Sulfate Roasting of Spent Lithium‐Ion Batteries
Source: Glob Chall. 2022 Nov 18;6(12):2200053. doi: 10.1002/gch2.202200053 (PMC9749078; doi:10.1002/gch2.202200053)
Supplement: Supplementary file 1 — Supporting Information [file GCH2-6-2200053-s001.pdf]

Global Challenges Open Access

Supporting Information

for *Global Challenges,* DOI: 10.1002/gch2.202200053

Mechanisms of the Ammonium Sulfate Roasting of Spent Lithium-Ion Batteries

*Xin Qu, Yiqi Tang, Mengting Li, DongXu Liu, Shuaibo Gao, and Huayi Yin**

Supporting Information

Mechanisms of the ammonium sulfate roasting of spent lithium-ion batteries

Xin Qu[1], Yiqi Tang[2], Mengting Li[2], DongXu Liu[2], Shuaibo Gao[1], Huayi Yin[1,2]*

1. School of Resource and Environmental Sciences, Wuhan University, 299 Bayi Road, Wuchang District, Wuhan 430072, P. R. China.

2. Key Laboratory for Ecological Metallurgy of Multimetallic Mineral of Ministry of Education, School of metallurgy, Northeastern University, Shenyang, P. R. China, 110819

Email: yinhuayi@whu.edu.cn

Data processing and Analyzing

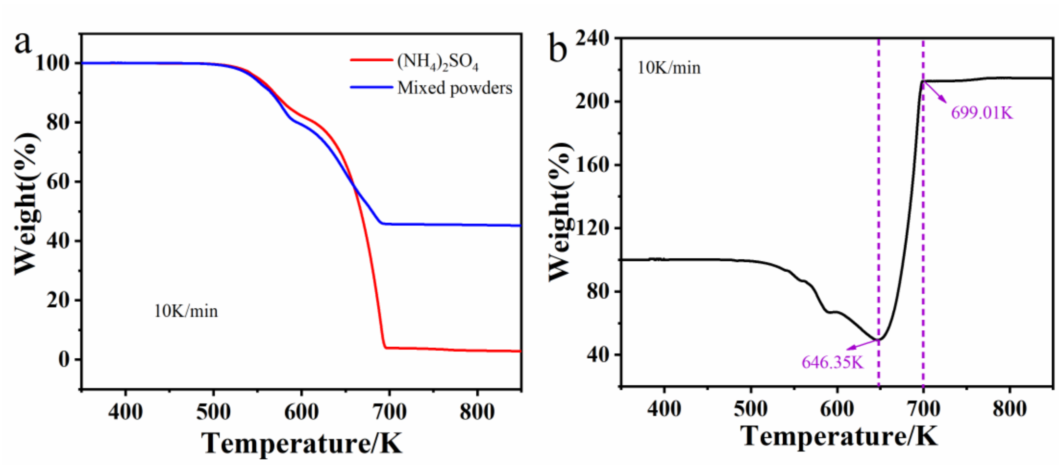

Figure S1. TG curves of pure $(NH_4)_2SO_4$ and mixed powders; TG curves of real LCO sulfation process. (atmosphere: Ar, heating rate: 10 K/min, $(NH_4)_2SO_4$/LCO=4:1 wt/wt)

$$T_1 = \frac{\Delta w_1}{w_1} \quad (S1)$$

$$T_2 = \frac{\Delta w_2}{w_2} \quad (S2)$$

$$T_3 = \frac{\Delta w_3}{w_3} \quad (S3)$$

$$\frac{w_1}{w_2} = 4:1 \quad (S4)$$

$$w_1 + w_2 = w_3 \quad (S5)$$

$$\Delta w_1 + \Delta w_2 = \Delta w_3 \quad (S6)$$

T_1, T_2 and T_3 represented TG changes of $(NH_4)_2SO_4$, LCO sulfation and mixed powders; w_1, w_2 and w_3 represented initial masses of $(NH_4)_2SO_4$, LCO sulfation and mixed powders; Δw_1, Δw_2 and Δw_3 represented mass changes of $(NH_4)_2SO_4$, LCO sulfation and mixed powders. Herein, T1 and T3 were obtained in **Figure S1**a by TG results. The mass ration of LCO and $(NH_4)_2SO_4$ was 4:1. And the relationship of TG changes of them could be illustrated as **Equation S4-S6**. At last, the TG change of LCO sulfation process as **Equation S2** can be deduced as **Equation S7**:

$$T_2 = \frac{\Delta w_2}{w_2} = \frac{\Delta w_3 - \Delta w_1}{w_2} = \frac{\Delta w_3}{w_2/5} - \frac{\Delta w_1}{\frac{w_1}{4}} = 5T_3 - 4T_1 \quad (S7)$$

After data processing, a real TG change of LCO sulfation process was obtained as **Figure S1**b. It could be observed that the weight loss occurred before the LCO sulfation process. Besides, the decomposition of $(NH_4)_2SO_4$ in mixed poders was sligthed increased because of the existence of LCO powders. As the reaction progressed, the generation of metal sulfation could delay the decomposition of $(NH_4)_2SO_4$. Moreover, temperature interval of LCO sulfation process ranged from 646.35K to 699.01K, which was also consistent with the results of roasting experiments. And the actual weight change (212.87%) was closed to the theoretical increase (224.49%). It could be lnβlnβsummarized that the determination of LCO sulfation process was reasonable in

the approximative case.

Besides, TG curve of pure$(NH_4)_2SO_4$ was adjusted by **Equation S7**. As shown in **Figure S2**, the decomposition of $(NH_4)_2SO_4$ in mised powders showed similarity to that of pure $(NH_4)_2SO_4$, which meant that decomposition properties of $(NH_4)_2SO_4$ were not substantially changed. Therefore, $(NH_4)_2SO_4$ could represent the decomposition behavior of $(NH_4)_2SO_4$ in mixed powders.

$$T_1' = \frac{\Delta w_1}{w_3} = \frac{\Delta w_1}{5/4 \times w_1} = \frac{4}{5} T_1 \quad \text{(S7)}$$

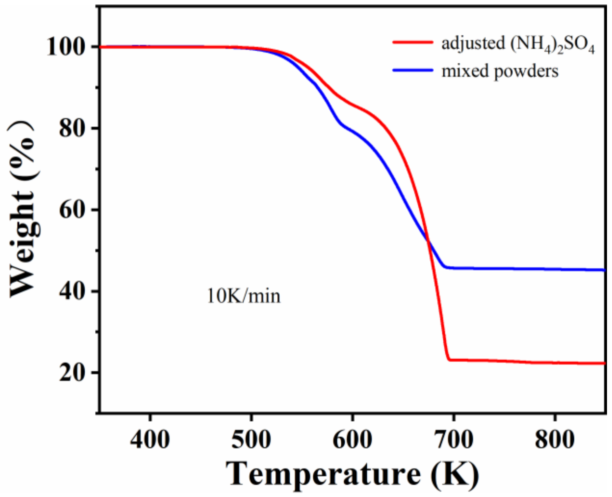

Figure S2 TG curves of adjusted pure $(NH_4)_2SO_4$ and mixed powders.

Table S1 Usual reaction mechanism function of solid phase

No.	Reaction model	Symbol	f(α)	g(α)
1		F1	(1-α)	-ln(1-α)
2	Chemical reaction	F3/2	$(1-\alpha)^{3/2}$	$2[(1-\alpha)^{-1/2}-1]$
3		F2	$(1-\alpha)^2$	$(1-\alpha)^{-1}-1$
4		D1	1/2α	α
5		D2	1/[-ln(1-α)]	(1-α)ln(1-α)+ α
6	Diffuse	D3	$3(1-\alpha)^{1/3}/2[(1-\alpha)^{-1/3}-1]$	$[1-(1-\alpha)^{1/3}]2$
7		D4	$3/2[(1-\alpha)^{-1/3}-1]$	$(1-2\alpha/3)-(1-\alpha)^{2/3}$
8		R1	1	α
9	Interface reaction	R2	$2(1-\alpha)^{1/2}$	$[1-(1-\alpha)^{1/2}]$
10		R3	$3(1-\alpha)^{2/3}$	$[1-(1-\alpha)^{1/3}]$
11		A2	$2(1-\alpha)[-\ln(1-\alpha)]^{1/2}$	$[-\ln(1-\alpha)]^{1/2}$
12	Nucleation and growth	A3	$3(1-\alpha)[-\ln(1-\alpha)]^{2/3}$	$[-\ln(1-\alpha)]^{1/3}$
13		A4	$4(1-\alpha)[-\ln(1-\alpha)]^{3/4}$	$[-\ln(1-\alpha)]^{1/4}$

Table S2 Calculation results of E and R^2 based on Šatava-Šesták method

Symbol	5 K min^{-1}		10 K min^{-1}		15 K min^{-1}		20 K min^{-1}		Average value	
	E (kJ mol^{-1})	R^2	E (kJ mol^{-1})	R^2	E (kJ mol^{-1})	R^2	E (kJ mol^{-1})	R^2	E (kJ mol^{-1})	R^2
F1	466.69	0.9895	371.44	0.9922	424.54	0.9903	339.69	0.9859	400.59	0.9895
F3/2	551.2	0.9856	436.3	0.9761	497.25	0.969	396.76	0.9591	470.38	0.9727
F2	649.35	0.9674	511.48	0.9488	581.37	0.936	462.69	0.9213	551022	0.9434
D1	681.06	0.9483	548	0.9731	629.93	0.9831	506.67	0.9897	591.41	0.9735
D2	748.81	0.9665	600.62	0.9851	689.25	0.9917	553.56	0.9952	648.06	0.9846
D3	97.64	0.9887	76.94	0.9705	87.41	0.9564	69.61	0.9429	82.9	0.9647
D4	72.76	0.8028	55.98	0.7484	62.74	0.7161	49.37	0.6874	60.21	0.7387
R1	340.53	0.9483	274	0.9731	314.96	0.9831	253.33	0.9897	295.71	0.9735
R2	396.79	0.9759	317.59	0.9902	364.04	0.9944	292.07	0.9958	342.62	0.9891
R3	48.82	0.9887	38.47	0.9705	43.7	0.9564	34.81	0.9429	41.45	0.9647
A2	233.35	0.9895	185.73	0.9922	212.27	0.9903	169.84	0.9858	200.3	0.9895
A3	466.7	0.9895	371.46	0.9922	424.53	0.9903	339.68	0.9858	400.59	0.9895
A4	116.67	0.9895	92.87	0.9922	106.13	0.9903	84.92	0.9858	100.15	0.9895
